# Supplementary material for: Parental Experiences of Melatonin Administration to Manage Sleep Disturbances in Autistic Children and Adolescent in the UK
Source: Healthcare (Basel). 2023 Jun 16;11(12):1780. doi: 10.3390/healthcare11121780 (PMC10298472; doi:10.3390/healthcare11121780)
Supplement: Supplementary file 1 [file healthcare-11-01780-s001.zip › healthcare-2315122-supplementary.pdf]

healthcare MDPI

Supplementary Materials

Topic Guide

1. Please tell me about you and your child's experience with sleep disorders and Autism Spectrum Disorder.
   - How is their sleep affected?
   - How does this impact their/your day?
   - How long has this been occurring?

2. What is your understanding of melatonin for treating sleep disorders?
   - What do you think it does?
   - How might it be useful?

3. What was your reason for starting to use melatonin?
   - Were you recommended it by your GP?
   - Did you read about it online?
   - Were you recommended by a friend or family member?

4. What was your child's response/reaction to using melatonin?
   - Did they react positively or negatively?
   - Did you/they find it to positively impact sleep?

5. What was/is your reasoning for no longer giving your child melatonin?
   - Did your child have any side effects? If so, what?
   - Were there any difficulties associated with using melatonin?

6. How did you use melatonin?
   - How much melatonin did you give?
   - What times did you give it?

7. What instructions were you given for using melatonin?
   - Light therapy?
   - Time of day?
   - Were you offered any alternatives to melatonin?

8. Did using melatonin resolve your child's sleep issues?
   - Do you have any positive experiences of using it?
   - Do you have any negative experiences of using it?

9. Did you find any drawbacks of using melatonin?
   - Did anything make it difficult to use?
   - Did it work in the way that you expected it to?

10. Overall, how would you describe your experience with using melatonin?
    - What are the most prominent factors that come to mind?
    - Would you say you had an overall positive or negative experience?

11. Would you recommend the use of melatonin to someone in a similar situation?
    - If so, why?
    - Did you have any concerns about giving your child melatonin?
